# The Effect of Wind Speed on Male Potato Aphid, *Macrosiphum euphorbiae*, Responses to Primary Host Plant Volatiles and Female Sex Pheromone

**DOI:** 10.3390/insects13040312

**Published:** 2022-03-23

**Authors:** W. Marie Alexander, Benjamin D. Rubin, Jeremy N. McNeil

**Affiliations:** Department of Biology, Western University, London, ON N6A 5B7, Canada; wmalexan@gmail.com (W.M.A.); brubin2@uwo.ca (B.D.R.)

**Keywords:** potato aphid, male response, host plant volatiles, sex pheromone, wind speed

## Abstract

**Simple Summary:**

We demonstrate that male potato aphids respond more to a combination of volatiles from the host plant on which the species overwinters and the female sex pheromone than to the host plant alone. In both cases, the level of response declines as wind speed increases, but the higher attraction to the combined odour sources is maintained. These findings are discussed within the context of mate location by male aphids, which are insects that have little control over flight direction.

**Abstract:**

In fall, alate males of the potato aphid, *Macrosiphum euphorbiae* (Thomas), migrate from their summer (secondary) host plants, such as potatoes, to primary host plants, such as roses, where they mate with wingless oviparae who produce the overwintering egg stage. Males are weak fliers and generally walk towards a pheromone source under windy conditions, so we tested the hypothesis that upwind walking behaviour in response to wind velocity would be affected by the volatile cues present. We compared male responses to the odour of a rugosa rose cutting alone and to the combination of host plant volatiles and the female sex pheromone under a range of wind speeds in a laboratory walking bioassay. The proportion of males responding decreased as the wind speed increased, but at all wind velocities, the responses to the combined odours were higher than to the host plant alone. However, at any given wind velocity, the speed at which responding aphids moved was not influenced by the odour source. These findings support the idea that host plant volatiles serve as long-distance cues for males and that the female sex pheromone is used once on the host plant.

## 1. Introduction

Many aphid species are dioecious, reproducing asexually through parthenogenesis in the summer on different plant species referred to as secondary hosts [1]. However, in response to cues such as decreasing temperatures, shortening day length, and deteriorating host plant quality, they switch to sexual reproduction in the fall [1]. At this time sexual morphs, the gynoparae (winged asexual females that produce the apterous, egg-laying oviparae) and males migrate to overwinter as diapausing eggs on different plant species known as primary hosts [1]. While both morphs are very weak fliers and have very little control over their flight direction [1], there is evidence that they use host plant volatiles to locate their primary hosts [2,3,4]. Furthermore, female sex pheromones serve as a cue for males to locate potential mates [2,4,5]. 

Wind velocity can modify the plume structure and concentration of infochemicals [6], as well as the calling behaviour of insects [7]. Furthermore, in the cases of weak flying insects, such as aphids, wind speeds above 2 m/s inhibited the flight of male aphids [8,9]. 

There has been little research examining the effects of wind velocity on the response of male aphids to primary host plant volatiles, with or without the presence of the female sex pheromone. Therefore, we tested the hypothesis that the impact of wind velocity on male mate-searching behaviour would be affected by the type of volatile cues present. We predicted that while responses would go down as wind velocity increased, males would exhibit stronger levels of response to both a primary host plant volatile and the female sex pheromone than to host plant volatiles alone. We used a wind tunnel walking assay and chose the diecious potato aphid *Macrosiphum euphorbiae* (Thomas) as previous research has shown that, in this species, both female calling behaviour and male response to the pheromone are affected by wind speed [9,10]. Furthermore, at a constant wind speed of 0.4–0.6 m/s, males showed a significantly higher response to volatiles from *Rosa rugosa* (rose) cuttings than to those from *Solanum tuberosum* (potato, their secondary host), but that their response to rose volatiles alone, or rose volatiles and pheromones, did not differ significantly.

## 2. Materials and Methods

### 2.1. Insects 

The aphids came from a laboratory colony established using field-collected individuals from fields near Quebec City and had been in continuous rearing for approximately three years. To ensure continuous asexual reproduction, the colony was maintained on potato seedlings, *Solanum tuberosum* c.v. Norland, at 21 ± 1 °C, 60 ± 10% relative humidity (RH) under a 16L: 8D photoperiod. New plants were provided twice a week. Sexual morphs were obtained by rearing apterous aphids in individual plastic cages (5 cm × 9 cm) at 18 ± 1 °C, 60 ± 10% RH under a 12L:12D photoperiod. Early instar nymphs were sorted by sex and reared in separate chambers to ensure that females were virgins and that males were not exposed to the sex pheromone prior to the time of testing. Each day, newly moulted adult oviparae and males were collected and held in separate cages to ensure that they were of known age when used in different experiments. 

### 2.2. Bioassays 

All assays were conducted in a laminar airflow wind tunnel (140.8 cm long × 64.8 cm wide × 64.8 cm high) located in an environmental chamber at 21 ± 1 °C and 60 ± 10% RH. Males were tested individually to either the odour of *Rosa rugosa* (rose) sprigs alone, or combined with a female sex pheromone source, at wind speeds of 0–4 m/s (at 0.5 m/s increments), with four replicates (12 individuals per replicate) at each wind speed. All assays were conducted during the 3rd to 6th hour of the photophase, the period of maximum calling activity by females under controlled laboratory conditions [10]. As our previous experiments have shown that males do not move upwind when there are no olfactory cues, this treatment was not included as a control.

In each assay, a 10 cm rose sprig was taken from plants grown in a greenhouse at 24 ± 1 °C under natural photoperiodic conditions and placed on a platform located 20 cm upwind of a second platform [11]. As previous studies have shown that males generally do not fly upwind to a pheromone source in a wind tunnel [9,10,11], the platforms were connected by a string bridge, and aphids were released at the 10 cm mid-point so that individuals could move up- or downwind. Individuals were given 180 s to respond to the test stimulus as our previous studies had shown minimal responses with longer exposure times. Individuals were only used once, and a new host plant sprig was used for each replicate. 

Initially, for the response of males to *R. rugosa* sprigs in combination with the female sex pheromone, we used a 6–8-day-old virgin oviparous female that once transferred onto the plant with a fine camel-hair paintbrush and exhibited overt calling behaviour: where the abdomen and hind legs were raised off of the plant [10]. However, at wind speeds of >1 m/s the movement of the plant sprig often caused the oviparae to stop calling. Therefore, we conducted a preliminary assay where individual 1–3-day-old naive males were exposed to either a 6–8-day-old calling female or a 3:1 synthetic blend of nepetalactol (3 uL) and nepetalactone (1 uL), a ratio similar to that emitted by 6–8-day-old oviparae [12] at a wind speed of 1 m/s (3 replicates of 12 individuals). As neither the proportion of males reaching the host plant (F_(1,4)_ = 0.022, *p* = 0.890) nor the time taken for males to reach either odour source (F_(1,4)_ = 0.023, *p* = 0.886) differed, we used the synthetic pheromone source placed in the rose cutting for all wind speeds >1 m/s. The synthetic sex pheromone blend was applied to a 5 cm diameter filter paper and replaced every 15 min. 

For all assays, the proportion of individuals reaching the source, and the time it took them to do so, were recorded. In addition, we compared the distance individuals moved (grouped into 2 cm increments) to the different odour sources at wind speeds from 1.5 to 4 m/s but not at lower wind speeds as there was not a significant effect on male responses. 

### 2.3. Statistics 

We conducted two-factor ANOVAs with odor source, wind speed, and their inter-action term as the independent variables. In each ANOVA, the unit of analysis is a replicate of 12 individuals. We considered four response variables: (1) the proportion of males reaching the odor source, (2) the proportion of males responding to the odor source (i.e., moving toward the odor source, regardless of whether they ever reached it), (3) the average distance travelled toward the odor source (calculated based on individuals who responded to the source), and (4) the average time to reach the odor source (calculated based on individuals who reached the source). The proportion reaching the odor source was analyzed separately for oviparous female experiments (wind speed ≤ 1 m/s) and for synthetic pheromone blend experiments (wind speed ≥ 1.5 m/s). Proportion responding and distance travelled were only analyzed for synthetic pheromone blend experiments. Proportional response variables were analyzed using an arcsine square root transformation. We inspected plots of residuals vs. fitted values and normal quantile—quantile (QQ) plots to ensure that ANOVA assumptions were reasonable in all cases. Statistical analyses were performed in R [13].

## 3. Results

**Figure 1:**
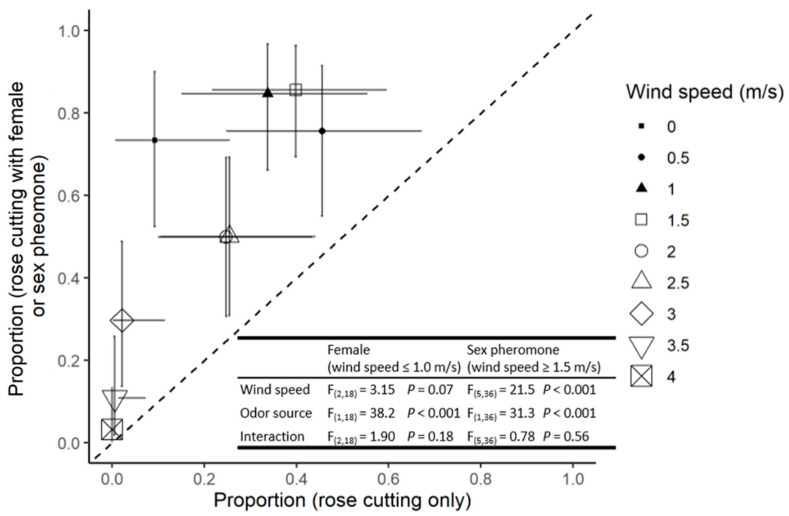
The proportion of males reaching the rose cutting (*x*-axis) and rose cutting with oviparous female (filled symbols) or sex pheromone blend (open symbols; *y*-axis). Symbols indicate the mean of four replicates and error bars indicate 95% confidence intervals. Points above the dotted 1:1 line indicate a preference for the female or pheromone treatment relative to rose cutting only. *F* statistics and *p* values are presented for the relevant ANOVAs.

At wind velocities between 0–1 m/s, there was no significant effect of wind speed on the proportion of males reaching a given odour source. However, significantly more individuals responded to the combination of the primary host volatiles and a calling female (Figure 1). 

At wind speeds between 1.5 and 4 m/s, there was a significant reduction in the proportion of males responding to either odour source as wind velocity increased. In addition, at any given wind velocity, the responses were significantly higher with the combined plant–pheromone blend than to the plant alone (Figure 1). At 4 m/s, only a few males responding to the combined volatiles from the rose and sex pheromones successfully reached the odour source (Figure 1). 

**Figure 2:**
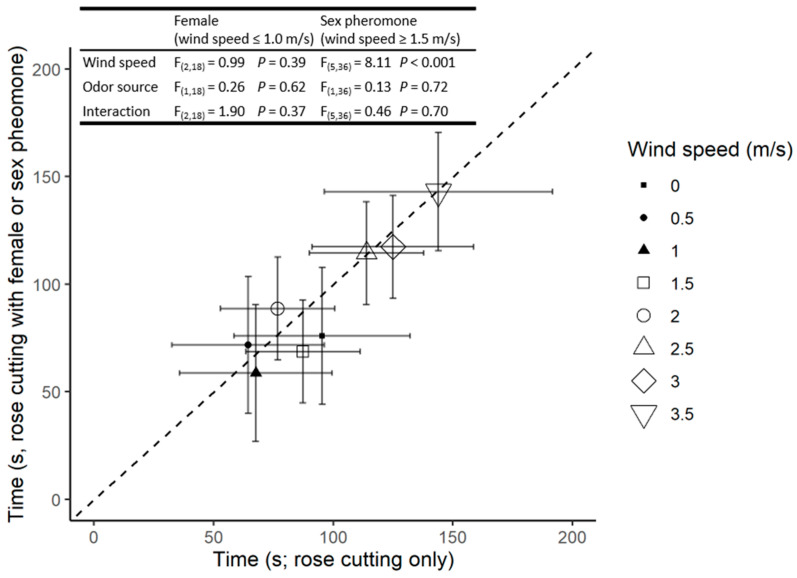
The time to reach the rose cutting (*x*-axis) and rose cutting with oviparous female (filled symbols) or sex pheromone blend (open symbols; *y*-axis). Symbols indicate the mean of four replicates based only on males who reached the odor source; and error bars indicate 95% confidence intervals. Points below the dotted 1:1 line indicate a preference for the female or pheromone treatment relative to rose cutting only. *F* statistics and *p* values are presented for the relevant ANOVAs.

There was also a significant increase in the time taken for responding males to reach the source as wind speed increased; however, there was no significant difference in response between the odour sources at any given wind velocity (Figure 2). 

**Figure 3:**
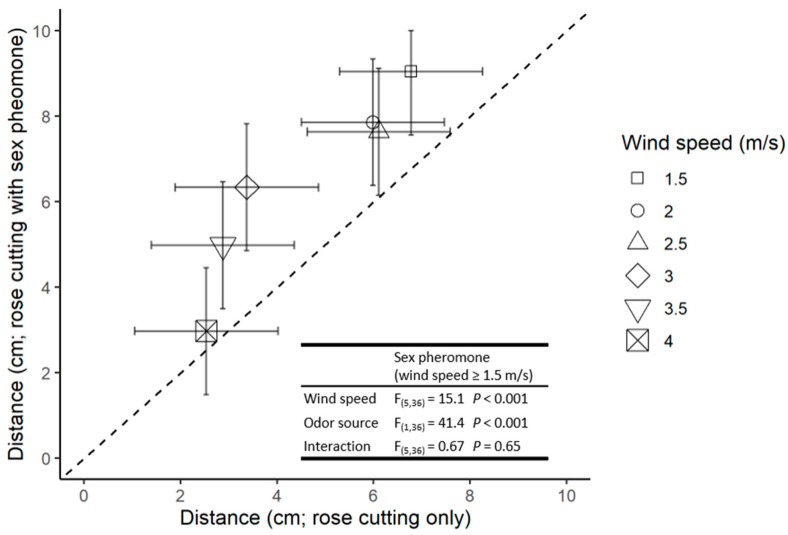
The distance travelled by males responding to the rose cutting (*x*-axis) and rose cutting with (*y*-axis). Symbols indicate the mean of four replicates and error bars indicate 95% confidence intervals. Points above the dotted 1:1 line indicate a preference for the pheromone treatment relative to rose cutting only. *F* statistics and *p* values are presented for the relevant ANOVAs.

At wind velocities >1.0 m/s, the overall distance males moved upwind to either odour source decreased significantly as the wind speed increased. However, males moved further upwind in the presence of the host plant and pheromone cues than to the host plant alone (Figure 3). 

## 4. Discussion

Our results confirm that the mate-searching behaviours of male potato aphids decrease as the wind speed increases. However, responses are higher when both primary host plant odour and female sex pheromone are present than with primary host plant volatiles alone, supporting the hypothesis that the impact of wind will, at least in part, be modulated by the olfactory cues present. Walking upwind at high wind speeds is not only energetically costly but also increases the probability of being blown off the rose plant [14], so given the trade-offs, males should be more likely to take a risk in the presence of a reliable cue (the pheromone) indicating the availability of a potential mate. The trade-off between undertaking a risky behaviour and the probability of acquiring a mate has been shown for other insects [15,16]; for example, more male moths flew upwind towards a high-quality pheromone source in the presence of auditory cues indicating the presence of a predator than when the pheromone source was of lower quality [16]. 

In a previous study on the response of male potato aphids at wind speeds of 0.4–0.6 m/s, the addition of the pheromone to the rose volatiles increased the proportion of male responding, but the effect was not statistically significant [11]. In our study, under the same conditions, the addition of the sex pheromone significantly increased the proportions of males reaching the source, but the response we observed to rose volatiles alone was lower than in the previous study. One possible explanation for this difference could be the quality of the rose clippings used, as the current study was carried out over a longer period while the plants were grown under the same greenhouse conditions, and the seasonal changes in natural light conditions that may have changed the volatile profiles in some way [17]. 

Males of *M. euphorbiae* significantly preferred the volatiles from the rose, the primary (overwintering) host, than those of the potato, a secondary (summer) host plant, on which they developed [11], supporting the hypothesis that they utilize primary host plant cues during migration from secondary to primary hosts. The initiation [18] and termination [19] of flight by aphids are active decisions, but once aloft, flight direction is determined mainly by wind currents [1,20]. Therefore, unlike strong flying insects, such as a male moth responding to a calling female [21,22], male aphids generally do not exhibit directed upwind flight towards an odour source [1]. They rely on cues, such as parts of the long-wavelength light spectrum reflected from both the ground and vegetation [1,23], and a specific blend of volatiles rising upwards on thermals from their host plants [24,25,26,27] to stimulate the active landing process. Consequently, the ability of an aphid to locate its specific host plant will, in large part, be determined by local abundance [26], as high densities of hosts would provide stronger sources of olfactory cues to initiate the landing process. 

Hurley et al. [11] proposed that male potato aphids would use the volatiles from a suitable primary host source (a large plant) to initiate landing and that the sex pheromone emitted by a calling virgin oviparous female (an organism <4 mm) would serve as a short-distance cue when searching for potentially receptive mates [9,11,28]. Our results support the idea that the presence of the female pheromone combined with primary host plant volatiles influence male mate-searching behaviour. If, as postulated, males use the sex pheromone as a foraging cue once on the primary host, then prevailing wind conditions would have an important impact on mate-searching behaviour. Not only would wind currents affect the dispersal of the odour plume but also the ability of the males to walk upwind to the source [9]. Our results suggest this is the case. For example, with respect to plume dispersal, one notes an increase in the response of males to rose volatiles as the wind velocity increases from 0–1 m/s, probably due to a higher number of molecules hitting the antennal receptors in a given time period [29,30]. However, as wind speeds increase from 1.5 to 4 m/s, there is a significant decrease in both the proportion of males reaching the source (Figure 1) and the distance they walked upwind (Figure 2), as well as an increase in the time taken for successful individuals to reach the source (Figure 3). This data set parallels the inhibition of flight activity reported for other small, weak flying insects [9,30,31,32]. However, under most wind velocities the responses differed when the source was the host plant volatiles and pheromones rather than the host plant alone. 

It has been estimated that only 1% of aphids reach suitable host plants [33], so it could be argued that this alone would be a reliable indicator of male quality, with only high-quality individuals surviving long enough to do so. However, as aphid flight is passive and mostly determined by wind currents [1], chance would play an important role in locating a suitable primary host plant. Therefore, the possibility that a male’s ability to successfully walk upwind at higher wind speeds once on the host plant reflects his quality merits further attention, particularly as females exhibit mate choice when males are placed next to them on the primary host (McNeil et al., unpublished data). 

The ability of males to detect and respond to the volatiles of primary host plants may also play an important role in the reproductive isolation of sympatric aphid species as the sex pheromone of many aphid species is composed of the same two components, nepetalactol and nepetalactone [34,35,36]. It has been proposed that specific communication channels could be obtained by having different ratios of the two components [37,38,39]. For example, *Aphis fabae* has a 1:29 ratio of nepetalactol:nepetalactone [37] while the ratio from *Cryptomyzus* spp. is 30:1 [38]. However, the pheromone of some aphid species, such as *Brevicoryne brassicae* [40] and *Sitobion fragariae* [41], have been reported to produce only nepetalactone which would not provide a reliable species-specific signal. Furthermore, the ratio of the two compounds emitted may change as a function of female age [12,36], and males respond to a range of ratios [12,39]. Therefore, the higher response to host plant volatiles in combination with sex pheromones would support the hypothesis that the two odours combined provide a unique signal for each species [38] and that it could play a role in the reproductive isolation of some aphid species [38,42]. This possibility requires further research as the males of some host-switching species do not apparently respond to the volatiles of primary host plants [3,4,38]; although, as proposed above, the actual growing conditions of the plant may influence the responses observed. 

## 5. Conclusions

Prevailing wind conditions significantly impact the mate-searching behaviour that the male potato aphids exhibit, with the proportion of males responding, the distance they move, and the speed of displacement that decreases with increasing wind speed. However, when both host plant volatiles and female sex pheromones are present, males exhibit higher levels of responses than to the host plant volatiles alone, especially at higher wind speeds. Thus, males will take greater risks in the presence of a receptive mate and, if this reflects male quality, may impact female mate choice. 

## Data Availability

The data sets are available from the authors upon request.
